# Importance of Social- and Health-Related Problems: Do Spaniards Give Them the Significance They Actually Deserve?

**DOI:** 10.3390/ijerph16214090

**Published:** 2019-10-24

**Authors:** Francisco Alonso, Cristina Esteban, Andrea Serge, Macarena Tortosa

**Affiliations:** 1 DATS (Development and Advising in Traffic Safety) Research Group, INTRAS (University Research Institute on Traffic and Road Safety), University of Valencia, 46022 Valencia, Spain; cristina.esteban@uv.es (C.E.); andrea.serge@uv.es (A.S.); 2 PRECOVIR (Prevention of Risk Behaviour on the Road), INTRAS (Research Institute on Traffic and Road Safety), University of Valencia, 46022 Valencia, Spain; macarena.tortosa@hotmail.com

**Keywords:** social problem, road traffic injuries, communicable diseases, noncommunicable diseases, public health

## Abstract

Social and health problems imply an impact on society. The main objective of this study is to provide an overview of how Spanish people perceive cancer, terrorism, cardiovascular diseases, crime, AIDS, drugs, and traffic accidents, finding out whether they assess the importance of these issues in correspondence with their actual severity. The study used a full sample of 1206 Spaniards (51.6% females and 48.4% males) who responded to a computer-assisted telephone interviewing (CATI) survey on the significance of these social and health-related problems, assessed through a zero to ten Likert scale. The perceived severity of the problems was considered taking into account the official data of deaths reported by governmental institutions. For the comparison of mean values, the One-way Analysis of Variance (ANOVA) test was used. Results show high average values for all the problems. The most concerning elements are cancer (*M* = 9.28 ± 1.24) and terrorism (*M* = 9.22 ± 1.47). Cardiovascular diseases have the lowest scores (*M* = 8.29 ± 1.64). There is a good adjustment between real and subjective perception, but some issues are either underestimated or overestimated. Women assessed all of them as more important than men, and people over 65 gave all the issues more value than younger people. It is important that Spaniards understand the objective severity of these issues, thus allowing for more interventions by governments, education, and mass media.

## 1. Introduction

Noncommunicable and communicable diseases remain a major source of concern among governments due to their implications for public health. Communicable diseases are understood as those that can be transmitted directly or indirectly from a person to another, and normally they are caused by bacteria, virus, parasites, or fungi [1]. Some of the most common ones are lower respiratory tract infections, AIDS, diarrheal diseases, and tuberculosis.

According to the World Health Organization (WHO), communicable diseases have been decreasing in the world, except for lower respiration infections which are still the third most common cause of death, although there were fewer victims reported in 2015 than in 2000 [2]. Diarrhea and tuberculosis remain in the list, but AIDS was no longer listed in 2015. In spite of this, in 2018, 37.9 million people with AIDS were registered [3], a number particularly high in low- and middle-income countries.

In order for the HIV virus not to become AIDS, which is the last phase of this disease, it is necessary to receive the appropriate medication. Thus, prevention, early detection, and clinical monitoring are key to slowing its progress [4]. However, only 53% of people with HIV are receiving the corresponding treatment [5]. In Spain, the introduction and expansion of this virus happened at the end of the 1980s, representing the highest rates in Western Europe [6]. This situation led the Spanish Government to introduce multiple measures in order to stop this situation, which caused a notable decrease in infected people during the following years [7].

On the other hand, noncommunicable diseases are those that cannot be transmitted from one person to another. The four main types are cardiovascular disease, cancer, chronic respiratory disease, and diabetes [8]. External injuries caused by accidents, homicides, or suicides are also included in this group [9].

Noncommunicable diseases usually appear to be the principal cause of death in the whole world [10,11]. Indeed, cardiovascular diseases remain the most common cause of death, and, instead of decreasing, they are increasing. The WHO reports that, by the year 2015, 8.76 millions of people had died just from ischemic heart disease, while in the year 2000 there were 6.88 million deaths as a consequence of the same disease [2]. The main risk factors for developing cardiovascular diseases are smoking, hypertension, diabetes, cholesterol, obesity, and a sedentary lifestyle, which are increasingly common in the population [12,13].

The situation is the same for cancer-related diseases, which went from being the ninth to being the fifth main cause of mortality in the world [2]. The most common type of cancer varies substantially among countries. In high-income countries, lung, prostate, colorectal, and breast cancer stand out, while low- and middle-income countries have higher rates of stomach, liver, and esophageal cancer [14]. In Spain, lung cancer ranks fifth among the diseases that caused more deaths in 2018, only behind ischemic heart disease, Alzheimer disease, dementia, stroke, and Chronic Obstructive Pulmonary Disease (COPD) [15].

Additionally, as a noncommunicable disease, delinquency and crime are very worrying. There are 6.1 homicide victims per 100,000 population worldwide [16]. Most homicides happen in America (36%), Africa (31%), and Asia (28%), while the numbers are much lower in Europe (5%) and Oceania (0.3%) [17]. Regarding terrorism, the most affected countries are Iraq, Afghanistan, Nigeria, Syria, and Pakistan, with thousands of deaths each year [18]. In Europe, Spain has the highest number of fatalities recorded between 2000 and 2018 (268), followed by France (263) and the United Kingdom (121) [19].

On the other hand, drug abuse affects more than 35 million people worldwide [20]. The most consumed drug is cannabis, followed by opioids and amphetamines or stimulants [21]. In Spain, drug abuse has remained stable in recent years, with cannabis (17.1% of young adults) and cocaine (3% of young adults) among the most consumed substances [22]. Consumption is concentrated among adolescents and adults under 35 years of age, being more common among men than women [23].

In general, in comparison with the major causes of death in the world by the year 2000, a new noncommunicable disease, which is more related to injuries, emerged in 2015’s list: Road injury [24]. Traffic accidents are considered one of the main health problems that need to be intervened in society, since they are responsible for human casualties related to injuries and deaths, and for the economic losses they cause. The worldwide rates of road traffic accidents in metropolitan areas have risen substantially over the last two decades. For instance, in the European Union, a total of 1.2 million accidents occur every year, leading to more than 40,000 fatalities [2,25]. Furthermore, according to the WHO, the number of road traffic accidents was already expected to increase by 80% from 2000 to 2020 [26].

To summarize, the income of countries is another factor that must be taken into account, but, in any case, until now it seems that health policies have been working on infectious diseases (which have decreased in the number of deaths they cause worldwide). However, the situation looks more complicated for noncontagious affections or noncommunicable diseases, which include the autonomy and the implication of individuals themselves, e.g., by making lifestyle changes to reduce cardiovascular problems [12], or reducing the consumption of cigarettes, which has been found [27,28] to be related with cancer, or to improve education in order to reduce criminality rates [29] and traffic accidents [30].

These data tell us about the official institutions’ worries and expenditures, but what about the opinion of citizens? Does the population understand the importance of those health issues? As an example, regarding traffic accidents, even though the overall society understands the seriousness implied in them, there are studies showing how certain population groups underestimate the risk of suffering a traffic accident [8]. About this, it has been found that the population groups who suffer the highest number of accidents are the ones who also perceive the lowest risk in this type of events. This is the case of young drivers, who perceive fewer risks than those with older age [11,31], and the case of men, who, even though they do perceive risks in a similar way to women, are less worried about suffering a traffic accident [32]. The consequences of this underestimation are utterly concerning since men under 25 have three times more probabilities of dying in a traffic accident than women who are the same age [24].

Nevertheless, we must say that traffic accidents are not the only health issue that is not correctly perceived (understanding this as the real risk implication reported by the mortality numbers associated with each problem). Even more so if we take into account that the world has changed extremely throughout the last few years, and a huge quantity of phenomena has taken place, including social reorganizations (e.g., migration), and new challenges in the economic field [33].

As we can see, there are multiple health and social issues that should concern the population. However, it is known that society does not correctly assess the severity of some of them [34] since some are underestimated, while others are overestimated. For instance, the issues of terrorist attacks [35] or plane crashes [36] are both events that are way overestimated in comparison to their real probabilities of occurrence.

It should be pointed out that the assessments of each issue go through a series of cognitive bias determined by aspects such as culture, personal experience, and educational level. This series of elements related to social cognition are affected, or at least influenced, by mass media [37]. There are events covered by press and television for days, such as the ones we have mentioned before, and other ones that are not even mentioned. Many times, this is not determined by the objective risk of the event, but it is rather the media interest that determines the “incorrect” assessment of severity that society gives to the event. Moreover, the individual behavior becomes important too since it is known that actions are potentially influenced by perceptions [38].

Although it is true that globalization and the quick spread of information through social media allow people to understand (or misunderstand) the aforementioned issues, and research has found out that they are important for directing actions and efforts to intervene, this does not mean that society gives them the same importance as governments and researchers. Furthermore, the risk associated with the difference between perception and reality implies a challenge that needs to be studied and, eventually, overcome. So, how do people perceive these problems, especially when they directly concern the place where they live? And how do they deal with public health policies?

### 1.1. Study Framework

In most health problems, preventive strategies display a higher degree of effectiveness, and they are also cost-effective from a societal and economical perspective, since they are specifically designed to avoid fatalities. As an example, Road Safety Education and Road Safety Training are the most effective tools for the prevention of the occurrence of traffic accidents. Both of them are meant to work on the “human factor”, and other aspects that determine the behavior of drivers and road users [30]. However, the available reports on road accidents are still a source of concern, and, in some countries, their results indicate that these training elements have not succeeded in accomplishing the proposed objectives [39]. Why might this be? Is it the same for other health issues? In order to answer these questions, the reality of their practice should be examined. When considering, for instance, the case of road safety education, some disadvantages are noticeable, such as its modest implementation in educational institutions, its almost nonexistent implementation after the stages of primary and secondary education, its guidance methodology, its foundations, and the generalized lack of adequate assessment of the educational programs [30,40]. The need to explore these limitations in a general public health perspective guides our main framework.

This research comes from a larger body of investigation on the study of road safety education, which seeks to analyze and understand how the Spanish population perceives health and traffic issues [30]. Moreover, the research reported in this manuscript focuses on the assessment of the importance of different social and health problems to know the subjective opinion of the Spanish population. For a full review, please consult the original study carried out in Spain [41].

### 1.2. Objectives

Taking into account the aforementioned Alonso’s et al. research [41], this manuscript presents the results of its first category “Assessment of general aspects”, in which participants were asked about their perception and assessment of different health problems. The main purpose of this paper was to provide an overview on how the Spanish people perceive health problems in their society, such as cancer, terrorism, cardiovascular diseases, crime, AIDS, drugs, and accidents, and to compare such problems with the real seriousness reported by the official government media. Moreover, we also aimed at studying the difference in the perception of these health problems, through sex and age as sociodemographic variables.

## 2. Materials and Methods

### 2.1. Participants

The sample was collected through a Simple Random Sampling proportional to age, sex, region, and habitat of the Spanish population. The number of participants necessary to represent a margin error for the general data of ± 2.9, with a confidence interval of 95.5% in the most unfavorable case of p = q = 50%, was *n* = 1200. Interviews were completed by 1206 Spanish people aged over 14 years, and the sample was composed of 622 women (51.6%) and 584 men (48.4%). They participated voluntarily and anonymously, thus guaranteeing their privacy, and we emphasized that data would be used for statistical and research purposes only.

### 2.2. Procedure, Design, and Ethics

By means of a cross-sectional design, and following an exploratory and descriptive methodology, this study gathered the data using a survey conducted through computer-assisted telephone interviewing (CATI) in order to reduce interview length and minimize recording errors. A 50-case pilot version was carried out (they were not considered for the final analysis). After some adjustments, the final average duration of the survey was 27 minutes. To achieve the proposed objectives, the following items from that questionnaire were considered:

*Sociodemographic variables*. Age and sex.

*Social and health problems*. Evaluated by means of a subjective perception assessment scale, in which participants were given the following instruction: “Assess the importance you give to each of the following issues. Use a scale from 0 to 10, in which 0 means that it is unimportant and 10 means that it is very important: Cancer, terrorism, cardiovascular diseases, crime, AIDS, drugs, and traffic accidents”. These diseases and problems which cause the highest number of deaths in Spain were selected together with those with a relatively high media interest in the Spanish social media. The seven-item instrument features a Cronbach alpha of α = 0.81, a reliability index which is quite acceptable, and which would not improve by eliminating items. As for the validity of the content, this study had the approval of the Scientific and Research Ethics Committee for Social Science in Health of the University Research Institute on Traffic and Road Safety (INTRAS) at the University of Valencia, composed of researchers and experts in the field who valued positively the capacity of the questionnaire to evaluate all the proposed dimensions.

For this type of study, ethical approval and formal consent are not required. The described research did not require the official intervention of the Ethics Committee in Experimental Research (consultative and advisory body of the University of Valencia) as no personal data were used and participation was anonymous. However, the Research Ethics Committee for Social Science in Health of the University Research Institute on Traffic and Road Safety (INTRAS) at the University of Valencia was consulted, certifying that the research subject to analysis responds to the general ethical principles, currently relevant to research in Social Science, and issued a favorable opinion for it to be carried out in Spain.

### 2.3. Data Processing

Descriptive statistics and confidence intervals were obtained first and were used to ascribe the Spanish population to different groups depending on sex and age. To conduct the comparison of mean values, the One-way Analysis of Variance (ANOVA) tests were used, based on the General Linear Model, with α = 0.05 significance level. All statistical analyses were performed using the Statistical Package for the Social Sciences version 23.0 (IBM SPSS Statistics for Windows, Version 23.0, Released 2015. IBM Corp, Armonk, NY, USA). 

The scores on the subjective importance of social and health-related issues were compared with the objective data on the number of deceased people corresponding to each one of them. We analyzed the incidence of the reported problems in Spain both in 2003 and in 2017 (public data is available at the National Statistics Institute of Spain [42,43] and from the White and Black Book of the European Parliament [19]) in order to observe their evolution between the time of the data collection and the recently reported numbers. We expected that the impact of all these issues would have decreased by the year 2017, compared to 2003. We also expected that people would give average to high importance to every issue and that the order would not correspond to the issues’ real severity. Moreover, we were expecting that both young people and men would assess such severity as lower.

## 3. Results

Participants were older than 14, and most of the sample was between 40 and 65 (36.8%) and 20–39 (34.9%) years old. 33.4% of the sample had first-grade secondary education, 24.8% had second-grade secondary education, and 21.9% had university studies. The rest had primary education (16.1%) or no education (3.8%). Regarding the labor situation, 46.4% of participants were employed, 18.6% worked exclusively in household chores, 15.3% were retired, and 13.6% were students.

Table 1 shows the results for the general perception of the attributed importance of the different social and health problems assessed. Considering the 0–10 Likert scale of importance, the average values are high for all questions, as they were all around 8–9. The issues that generate the biggest concern in the subjects were cancer (*M* = 9.28 ± 1.24), terrorism (*M* = 9.22 ± 1.47), and traffic accidents (*M* = 8.68 ± 1.56), whose significance is assessed similarly to other problems, in order, drugs, AIDS, crime, and cardiovascular diseases (*M* = 8.29 ± 1.64).

Significant differences were found between the valuation of those problems *F* (6,1206) = 99.33, *p* < 0.001 (see Table 2). In this sense, cancer and terrorism obtained scores significantly higher than those of the rest of the problems, while cardiovascular diseases presented statistically lower assessments than the rest of them.

### Differential Analysis of Social and Health Problems Perception According to Sociodemographic Variables

Regarding the sex variable, statistically significant results were found in all the assessed problems. When compared with the assessments given by men, it was observed that women gave more importance to all of the problems (see Table 3). Moreover, it was found that women give cancer and terrorism the highest value of importance, despite the fact that men are the ones most affected by problems related to cancer.

Regarding the age of participants, the different age groups assess the importance of the various issues as high, showing significant differences between five of them (see Table 4). Older age groups (≥65 years and 40–64 years) are the ones that give the highest importance to all of these significant problems (see Table 5). 

With respect to the difference between the evaluations performed by the different age groups regarding the five issues that present significant differences, it was found that people over 65 perceive these problems as the most important ones (all but AIDS), with average values ranging between 8.55 ± 1.54 and 9.58 ± 1.01. In the case of AIDS, young people between 14 and 19 are the ones who give it the highest importance (*M* = 8.99 ± 1.27).

## 4. Discussion

This research aimed at providing an overview of the perception of the Spanish population concerning health and social problems by comparing them with their actual seriousness. This study shows that the Spanish population is actually concerned about health, and what is interesting is how they are concerned.

In Table 6, data on deaths in Spain that were caused by social and health problems are shown, taking into account sex and age, both in the year 2003 and in 2017; finding that the main causes of death in Spain follow the global tendency. Noncommunicable diseases, such as hypertension, are becoming the most important cause of mortality in this country [44]. Moreover, cardiovascular diseases and road traffic injuries are the leading causes regarding the years lived with a disability [45].

This allows us to recognize a decrease in the number of deaths in 2017 compared with 2003, with the exception of cancer and drugs, where the number of fatal victims has been increasing. Nevertheless, cardiovascular diseases are still the leading cause of death in the list for what concerns Spain. It is also noteworthy that AIDS and crime are not related to the highest number of deaths; there is indeed a noticeable reduction of their impact, as data from 2017 show. Additionally, the figures show age-related differences in the number of deaths, especially regarding cancer and cardiovascular diseases; here, it seems that to an older age corresponds a higher number of deaths, and, with the exception of cardiovascular diseases, more men die because of this than women [42,43].

Taking this into account, we compared the perception attributed by the Spanish population and the real or objective importance of these problems through the data reported by official entities. Our results suggest that Spanish people attribute the highest importance values to cancer, terrorism, and traffic accidents. The opposite happens for cardiovascular diseases and crime, which obtained the lowest average scores in the assessment. And finally, AIDS and drugs obtained intermediate values within the average.

Taking into account that the main cause of death in Spain, that is, the one with the highest number of deaths, both in the year 2003 and in 2017, corresponded to cardiovascular diseases, it is surprising that it is also the problem that was given the lowest importance by participants. The fact that it is the least valued issue may be related to the increasing trend in developed countries regarding tobacco consumption, physical inactivity, diabetes, and obesity, which are risk factors for cardiovascular disease [12].

Taking cancer into account, research shows that this group of diseases corresponds to the second main cause of death in Europe [3]. Moreover, trends show that it is the second main cause of death in Spain as well, both in 2003 and 2017. It is worrying that, despite its real and subjective importance, the number of victims related to this disease keeps increasing in Spain. A possible explanation of this phenomenon could be related to alcohol consumption, lifestyles, and smoking rates [45]. Furthermore, as our results show, men are less concerned about this problem than women, which could lead to a tendency to get involved in risky behaviors, not knowing their possible consequences. On the other hand, even though elderly people are the ones who die the most because of this disease, there are no significant differences in this Spanish people’s group.

Otherwise, it is worth noticing that a problem such as terrorism, which produces fewer victims than road traffic accidents [46], is considered a major problem. In spite of this general perception, the objective data for 2003 were three casualties, reduced to zero in 2017. It is true, however, that terrorism is a health problem in terms of injuries, which causes social and emotional damage, and that Spain has a long terrorism-related history, which easily explains its importance in this country [47,48]. Actually, there were terrorist attacks in the year 2000 (26 casualties), 2001 (15 casualties), and 2002 (5 casualties) [19]. This is attention-worthy, since, despite reporting fewer numbers in comparison with other problems, terrorism has an emotional and subjective component that allows for its high assessment. Additionally, there is no control perception in this type of event, and this affects the assessment of their severity. Moreover, in our results, we found that crime was poorly evaluated, and even though terrorism and crime are related to each other, the Spanish population evaluates them differently; it is clear that this is due to the media, which give more importance to terrorism and its consequences than to other types of outcomes which are statistically more worrying in terms of health [49].

On the topic of traffic accidents, their assessed importance obtained an average value of 8.68 + 1.56, and both men and women evaluated this topic significantly. Again, women assessed them as more important than men did. Regarding this, it is an extended belief that women can be more exposed to risk and are more vulnerable to injuries than men [50], even though the numbers show the contrary: More men die in traffic accidents than women, as data from both 2003 and 2017 show. These road injuries findings follow the trend of sex roles in traffic, where males present higher accident rates [51], as well as more sensation seeking [52].

On this point, Alonso, Montoro, Tortosa, and Martinez indicate that the analysis of traffic accidents should include not only the accidents themselves and the conditions directly related to them, but also the set of socioeconomic conditions, legislations, and interventions surrounding this phenomenon [53]. Finally, road accidents are recognized as one of the principal causes of posttraumatic stress disorder [54], as well as a social and health problem that affects families, communities, and societies [55].

It should be pointed out that the number of deaths on the road was lower in the year 2017 than in 2003, which could be associated with the inclusion of the points system in driver licenses, the relevant increase in traffic campaigns and the implementation of road safety education in schools, among others. Furthermore, Spain shows one of the lowest rates of accidents in Europe [56].

On the other hand, even though there are some differences between men and women, overall, both women and men think that all factors that were presented in this research are relevant, since all of them obtained scores above 8. However, we should not forget that women scored more than men in the whole survey. What is relevant about this is that there are more men who die in every single social and health problem, with the exception of cardiovascular diseases where deaths are more common in women, thus being the only problem that is more prevalent in women than in men. However, even though women give more importance than men (*M* = 8.58 ± 1.53 and *M* = 7.99 ± 1.69) to them, these values are still low in comparison with other results. It is clear that this whole situation constitutes a health problem that must be addressed urgently.

Regarding age, overall, every problem was assessed as more important by people over 65, and this could be related to their own health issues and to the perceptions proper to their particular stage of development [57]. AIDS is an exception to this since it is assessed as more important by people younger than 19, maybe because of their easier access to information and their better sex education. The differences between the perception of people between 20 and 64 are not so clear, despite representing the great majority of deaths for each one of the addressed issues (with the exception of cardiovascular diseases, which cause more deaths in people older than 65).

## 5. Conclusions

The conclusion from a practical point of view is clear: There is a high level of awareness about social and health problems among the Spanish population. This fact could be a facilitator for the development of new measures and countermeasures in order to help alleviate the social consequences of all these issues.

We can also conclude that we must keep working in order to increase awareness on the importance of health and social issues, especially on cardiovascular diseases, and also keep working on the responsibility of habits in their prevalence. In other words, we need to enhance the comprehension of what people could do in order to prevent all the social and health issues that we evaluated. This must be done in the general population with two clear objectives: To strengthen the right concepts related to illnesses and social problems, and to modify incorrect beliefs.

As for the initial hypothesis: (1) As expected, most problems have decreased the number of deaths related to them, except for drugs and cancer, which have increased. Cancer is particularly worrying, given that there is a large number of deaths related to it, and it is also assessed as very important by the population; however, it has not been possible to reduce its related deaths; (2) With the exception of two problems (cardiovascular diseases and terrorism), people estimate the objective importance of each problem quite correctly; (3) As it had been stated, men and young people give less importance to the evaluated problems, with the exception of AIDS.

As these results show, it is important to go in-depth into several social and health problems in Spain. The real concern of participants regarding these issues is the first step for intervention, since they are already part of the social representations of the Spanish people, considering the fact that when society agrees on the importance of a problem, it is easier for people to approve the solution and be part of it. As a second step, citizens, especially young people, must be made aware of the real importance of each one of the problems, and from there, effective prevention measures must be established.

In this sense, a responsible exercise from the government and the media would be to contribute to the education of society by providing information about the problems that have a greater objective risk [34]. Taking advantage of the fact that the subjective and the real assessments in Spain are rather similar, they should pay special attention and invest in studying these phenomena based on the evidence of underestimated and overestimated events, in order to achieve effective interventions.

On the other hand, it should be noted that, although the population perceives the problems presented as important, this is not a clear indicator that the institutions have acted on them, or that the actions that have been carried out are workable.

### Limitations and Future Research

Since this work was carried out using self-reports as the primary source of information, it is necessary to recognize that social biases such as desirability or a poor understanding of the questions could be present in our results [58]. Additionally, in order to avoid biases related to an unawareness of the issues, this research assessed general problems, which prevented us from knowing the subjective perception on more specific problems.

Simultaneous interviews might help with the understanding of the personal profiles of the participants. Moreover, the use of not just quantitative analysis but also qualitative analysis would allow us to comprehend why the Spanish population consider some problems to be more worrying than others. Additionally, it is important to emphasize the work on social representations and how they can be used in order to create new representations in the scope of public health.

Regarding future research, this study on perceptions allows us to elaborate three possible lines of research: (1) Even when communication about health issues appears to be the main goal of different governmental initiatives [59], what is the real effectiveness of the communication programs carried out in Spain?; (2) Do perceived health problems obey emergent problems, or do they obey certain social features of the Spanish population?; and (3) What is the perception of the Spanish people on specific problems contained in the general problems that have been assessed? For instance, what do they know about the different types of cancer? What do they perceive regarding risk factors affecting cardiovascular diseases? And so on and so forth.

## Figures and Tables

**Table 1:** Estimated importance of social and health problems by order of valuation.

Social and Health Problems	Attributed Importance			CI 95%	
	*M*	*SD*	Mdn	Lower	Upper
Cancer	9.28	1.24	10	9.21	9.35
Terrorism	9.22	1.47	10	9.14	9.31
Traffic Accidents	8.68	1.56	9	8.59	8.76
Drugs	8.58	1.99	9	8.47	8.70
AIDS	8.54	1.89	9	8.44	8.65
Crime	8.49	1.80	9	8.39	8.60
Cardiovascular Diseases	8.29	1.64	8	8.20	8.39
Cronbach alpha of α = 0.81					

**Table 2:** Post hoc analysis for inter-subject differences in the assessment of social and health problems.

Social and Health Problems			Bonferroni			
	*M*	*SD*	Cancer	Terrorism	Cardiovascular Diseases	Crime
Cancer	9.28	1.24			<0.001	
Terrorism	9.22	1.47			<0.001	
Cardiovascular Diseases	8.29	1.64	<0.001	<0.001		
Crime	8.49	1.80	<0.001	<0.001	0.006	
AIDS	8.54	1.89	<0.001	<0.001	<0.001	
Drugs	8.58	1.99	<0.001	<0.001	<0.001	
Traffic Accidents	8.68	1.56	<0.001	<0.001	<0.001	0.028
F(6,1206) = 99.33, *p* < 0.001						

**Table 3:** Valuation of importance and figures of social and health problems according to sex.

Health Problems	Attributed Importance				
	T Student	Men (*n* = 584)		Women (*n* = 622)	
	t(1204)	*M*	*SD*	*M*	*SD*
Cancer	−6.39 *p* < 0.001	9.05	1.29	9.50	1.15
Terrorism	−5.71 *p* < 0.001	8.97	1.55	9.45	1.35
Cardiovascular Diseases	−6.36 *p* < 0.001	7.99	1.69	8.58	1.53
Crime	−7.01 *p* < 0.01	8.13	1.82	8.84	1.69
AIDS	−7.97 *p* < 0.001	8.10	2.10	8.96	1.55
Drugs	−9.55 *p* < 0.001	8.03	2.24	9.10	1.56
Traffic Accidents	−6.93 *p* < 0.001	8.22	1.69	9.11	1.29

**Table 4:** Valuation of importance of social and health problems according to age.

Health Problems	Attributed Importance by Age							
	14–19 (*n* = 118)		20–39 (*n* = 421)		40–64 (*n* = 443)		≥65 (*n* = 223)	
	*M*	*SD*	*M*	*SD*	*M*	*SD*	*M*	*SD*
Cancer	9.14	1.10	9.23	1.26	9.35	1.14	9.34	1.45
Terrorism *	9.03	1.34	8.99	1.71	9.32	1.41	9.58	1.01
Cardiovascular Diseases *	7.76	1.63	8.13	1.70	8.47	1.58	8.55	1.54
Crime *	8.09	1.81	8.14	1.90	8.57	1.80	9.20	1.27
AIDS *	8.99	1.27	8.54	1.90	8.36	1.99	8.67	1.88
Drugs *	8.34	1.84	8.18	2.31	8.82	1.69	9.00	1.81
Traffic Accidents	8.50	1.42	8.65	1.52	8.77	1.58	8.63	1.65

* Significant results for *p* < 0.05.

**Table 5:** Post hoc analysis for age differences in the evaluation of social and health problems.

**Terrorism**			**Games-Howell**			
**Age**	***M***	***SD***	**14–19**	**20–39**	**40–64**	**≥65**
**14–19**	9.03	1.34				
**20–39**	8.99	1.71				
**40–64**	9.32	1.41		0.011		
**≥65**	9.58	1.01	0.001	<0.001	0.028	
Welch F(3,441.63) = 12.17; *p* < 0.001						
**Cardiovascular Diseases**			**Tukey**			
**Age**	***M***	***SD***	**14–19**	**20–39**	**40–64**	**≥65**
**14–19**	7.76	1.63				
**20–39**	8.13	1.70				
**40–64**	8.47	1.58	<0.001	0.012		
**≥65**	8.55	1.54	<0.001	0.011		
F(3,1201) = 9.21; *p* < 0.001						
**Crime**			**Games-Howell**			
**Age**	***M***	***SD***	**14–19**	**20–39**	**40–64**	**≥65**
**14–19**	8.09	1.81				
**20–39**	8.14	1.90				
**40–64**	8.57	1.80		0.004		
**≥65**	9.20	1.27	<0.001	<0.001	<0.001	
Welch F(3,433.48) = 27.63; *p* < 0.001						
**AIDS**			**Games-Howell**			
**Age**	***M***	***SD***	**14–19**	**20–39**	**40–64**	**≥65**
**14–19**	8.99	1.27				
**20–39**	8.54	1.90	0.015			
**40–64**	8.36	1.99	<0.001			
**≥65**	8.67	1.88				
Welch F(3,465.92) = 6.03; *p* < 0.001						
**Drugs**			**Games-Howell**			
**Age**	***M***	***SD***	**14–19**	**20–39**	**40–64**	**≥65**
**14–19**	8.34	1.84				
**20–39**	8.18	2.31				
**40–64**	8.82	1.69		<0.001		
**≥65**	9.00	1.81	0.008	<0.001	<0.001	
Welch F(3,424.67) = 11.11; *p* < 0.001						

**Table 6:** Deaths due to social and health problems in Spain in 2003 and 2017.

**Health Problems**	**Number of Casualties 2003 ^1^**				**Number of Casualties 2017 ^1^**			
	**Total**		**Men**	**Women**	**Total**		**Men**	**Women**
Cancer	99,826		62,326	37,500	113,266		68,508	44,758
Terrorism ^2^	3		-	-	0		0	0
Cardiovascular Diseases	129,783		58,726	71,057	122,466		56,180	66,286
Crime	446		316	130	325		207	118
AIDS	1632		1289	343	442		342	100
Drugs	523		440	83	759		493	266
Traffic Accidents	5514		4240	1274	1943		1507	436
	**Number of Casualties 2003 by Age**				**Number of Casualties 2017 by Age**			
	**0–19**	**20–39**	**40–64**	**≥65**	**0–19**	**20–39**	**40–64**	**≥65**
Cancer	349	1777	22,122	73,792	264	1127	25,417	86,458
Terrorism ^2^	-	-	-	-	0	0	0	0
Cardiovascular Diseases	104	939	10,693	118,057	82	545	9962	111,877
Crime	20	222	152	52	24	108	129	64
AIDS	12	739	804	77	0	24	352	58
Drugs	4	350	395	16	1	160	316	282
Traffic Accidents	632	2266	1485	1131	112	555	741	535

^1^ Public data available at the National Statistics Institute [42,43] and from the White and Black Book of the European Parliament [19]; ^2^ There are no specific data for age comparison from 2003 (*n* = 3). Data from 2016 were taken into account, due to the lack of data from the year 2017.

## Data Availability

The datasets used and/or analyzed during the current study are available from the corresponding author on reasonable request.
